# Study analytical function subordination properties by applying a novel linear operator

**DOI:** 10.12688/f1000research.174492.1

**Published:** 2025-12-31

**Authors:** Maryam S. Majel, Mustafa I. Hameed

**Affiliations:** 1 University of Anbar, Ramadi, Al Anbar Governorate, Iraq; 2 University of Anbar, Ramadi, Al Anbar Governorate, Iraq

**Keywords:** Univalent Function, Best Dominant, Derivative Operator, Differential Subordination, Convex Function, Hadamard Product.

## Abstract

**Background:**

The study of theory for analytic univalent and multivalent functions is an old subject in mathematics, particularly in complex analysis, that has captivated a great deal of scholars owing to the sheer sophistication of its geometrical features as well as its many research possibilities. The study of univalent functions is one of many significant elements of complex analysis for both single and multiple variables. Investigators have become keen on the conventional investigation of this topic since at least 1907. Numerous scholars in the area of complex analysis have emerged since then, including Euler, Gauss, Riemann, Cauchy, and other people. Geometric function theory combines geometry and analysis.

**Methods:**

This study employs the differential subordination technique to derive multiple characteristics from the new linear operator $M_{\sigma ,\mu }^{n,\varsigma }\Upsilon \left(s\right)$ . The concept of the differential subordination subclass of analytical univalent functions is analyzed.

**Results:**

In this section, We studied some results on differential subordination and superordination using a specific class of univalent functions stated on a specific space of univalent functions stated on the open unit disc. Using properties of the operator, we discovered a number of properties of superordinations and subordinations related to the idea of the Hadamard product. We investigated several aspects of superordinations and subordinations using a new operator $M_{\sigma ,\mu }^{n,\varsigma }\Upsilon \left(s\right)$ .

**Conclusions:**

A new operator $M_{\sigma ,\mu }^{n,\varsigma }\Upsilon \left(s\right):\Lambda ⟶\Lambda$ has been established in this paper connected to the Dziok-Srivastava operator $T_{\sigma }^{n}$ and the Hadamard product corresponding to the Komatu integral operator $\Omega_{\mu }^{\varsigma }$ . The difference operator $M_{\sigma ,\mu }^{n,\varsigma }ϒ\left(s\right)$ can have specific properties derived by applying the differential subordination technique. And the objective of this paper is to make use of the connection $\left(\beta_{1}\mu +1\right)M_{\sigma ,\mu }^{n+1,\varsigma }ϒ\left(s\right)=w{\left(M_{\sigma ,\mu }^{n,\varsigma }ϒ\left(s\right)\right)}^{\prime }+\beta_{1}\mu \left(M_{\sigma ,\mu }^{n,\varsigma }ϒ\left(s\right)\right).$

## 1. Introduction

Gronwall’s Area Theorem, published in 1914, was a significant contribution to the theory of univalent functions. It is used for getting bounds on the coefficient values for meromorphic functions. Bieberbach resolved a similar issue regarding the class in 1916, as well as his known conjecture, which was mentioned in the exact same year but not applied until 1984, influenced the development of different methods in the geometric theory of complex variable functions. Estimates on the first two Taylor-Maclaurin coefficients are common in the study of bi-univalent functions since they are in the classes investigated by Gronwall and Bieberbach. Littlewood ^1^ is the source of the fundamental findings on subordination that Rogosinski ^2^ created and established. Subordination was recently utilized by Srivastava and Owa ^3^ to investigate the fascinating properties of the generalized hypergeometric function. A paper on differential subordinations, a generalization of differential disparities, was written by Miller and Mocanu. ^4^ The article is mainly concerned with the differential superordination of univalent functions in an open unit disk. To determine subordination properties, utilize the capabilities of the recently introduced operator $M_{\sigma ,\mu }^{n,\varsigma }ϒ\left(s\right)$ . Miller as well as Mocanu created the differential subordinations method in 1978, and the theory started to take shape in 1981. Refer to ^5– 9^ for further information. Suppose Λ indicates the type of functions of the given form

$$ϒ\left(s\right)=s+\sum_{\eta =2}^{\infty }e_{\eta }s^{\eta },\;e_{\eta }\;\geq \;0,$$ (1)

is analytic function in unit disk $\Delta^{*}=\left\{s:s\;\in \;\mathbb{C},\left|s\right|<1\right\}$ . Allow ϕ in Λ be provided by

$$\phi \left(s\right)=s+\sum_{\eta =2}^{\infty }d_{\eta }s^{\eta },\;d_{\eta }\;\geq \;0.$$ (2)

The following is the Hadamard product of ϒ and ϕ :

$$\left(ϒ*\phi \right)\left(s\right)=z+\sum_{\eta =2}^{\infty }e_{\eta }d_{\eta }s^{\eta },e_{\eta }d_{\eta }\;\geq \;0.$$ (3)

When ϒ and ϕ are analytic in $\Delta^{*},$ the function ϒ is considered subservient to ϕ, expressed as

$$\;ϒ\left(s\right)\;\;≺\;\;\phi \left(s\right)\;\left(s\;\in \;\Delta^{*}\right).$$

The previously Schwarz function ω is present in $\Delta^{*}$ if and only if |w(ω)|<1 as well as ω(0)=0 , if and only if there exists the Schwarz function ω , in $\Delta^{*}$ , with ω(0)=0 and |w(ω)|<1 such that

$$ϒ\left(s\right)=\phi \left(\omega \left(s\right)\right),\;\left(s\;\in \;\Delta^{*}\right).$$

Additionally, we’re left with the following equivalency if ϕ is univalent in $\Delta^{*}$ by, ^10– 13^: ϒ≺ϕ if and only if ϒ(0)=ϕ(0) and $ϒ\left(\Delta^{*}\right)\;\subset \;\phi \left(\Delta^{*}\right).$

## 2. Definitions and Lemmas

Definition 2.1. ^14^ The definition of the Komatu Integral operator $\Omega_{\mu }^{\varsigma }:\Lambda \to \Lambda$ for ϒ∈Λ is

$$\Omega_{\mu }^{\varsigma }\left(s\right)=s+\sum_{\eta =2}^{\infty }{\left(\frac{\mu +\eta -1}{\mu }\right)}^{-\varsigma }s^{n},\;\left(\mu >0;\varsigma \;\geq \;0\right).$$ (4)

Definition 2.2. ^15^ The definition of the Dziok-Srivastava operator $\Omega_{\mu }^{\varsigma }:\Lambda \to \Lambda$ for ϒ∈Λ is

$$T_{\sigma }^{n}\left(\beta_{1},\beta_{2},..,\beta_{n};\pi_{1},\pi_{2}..,\pi_{\sigma }\right)ϒ\left(s\right)=s+\sum_{\eta =2}^{\infty }\frac{{\left(\beta_{1}\right)}_{\eta -1}{\left(\beta_{2}\right)}_{\eta -1}\ldots {\left(\beta_{n}\right)}_{\eta -1}}{{\left(\pi_{1}\right)}_{\eta -1}{\left(\pi_{2}\right)}_{\eta -1}\ldots {\left(\pi_{\sigma }\right)}_{\eta -1}}\left(\frac{\mu +\eta -1}{\mu }\right)e_{\eta }s^{\eta },$$ (5)

where $\beta_{n}\;\in \;\mathbb{C},n=1,2,3,\ldots ,n,\pi_{n}\;\in \;\mathbb{C}\setminus \left\{0,-1,-2,..\right\},n=1,2,..,\sigma .$

Definition 2.3. If a function ϒ(s)∈Λ and Hadamard products between operator $\;T_{\sigma }^{n}\;$ as well as operator $\Omega_{\mu }^{\varsigma }\;$ given a new linear operator $\;M_{\sigma ,\mu }^{n,\varsigma }ϒ\left(s\right):\Lambda \to \Lambda$ , define

$$\begin{array}{c} M_{\sigma ,\mu }^{n,\varsigma }ϒ\left(s\right)=T_{\sigma }^{n}ϒ\left(s\right)\;*\;\Omega_{\mu }^{\varsigma }\left(s\right),\;\left(s\;\in \;\Delta^{*}\right),\;\text{and}\\ M_{\sigma ,\mu }^{n,\varsigma }ϒ\left(s\right)=s+\sum_{\eta =2}^{\infty }\frac{{\left(\beta_{1}\mu^{\varsigma }\right)}_{\eta -1}{\left(\beta_{2}\right)}_{\eta -1}\ldots {\left(\beta_{n}\right)}_{\eta -1}}{{\left(\pi_{1}\right)}_{\eta -1}{\left(\pi_{2}\right)}_{\eta -1}\ldots {\left(\pi_{\sigma }\right)}_{\eta -1}}{\left(\frac{1}{\mu +\eta -1}\right)}^{-\varsigma +1}e_{\eta }s^{\eta }. \end{array}$$ (6)

Form Eq. 6, we have

$$\left(\beta_{1}\mu +1\right)M_{\sigma ,\mu }^{n+1,\varsigma }ϒ\left(s\right)=w{\left(M_{\sigma ,\mu }^{n,\varsigma }ϒ\left(s\right)\right)}^{\prime }+\beta_{1}\mu \left(M_{\sigma ,\mu }^{n,\varsigma }ϒ\left(s\right)\right),\;\left(s\;\in \;\Delta^{*}\right)$$ (7)

It should be noted that $M_{\sigma ,\mu }^{n,\varsigma }ϒ$ has the following special cases.

a) The Komatu Integral operator $\Omega_{\mu }^{\varsigma }$ should be included if n=1,σ=1 by. ^14^
b) Add the Dziok-Srivastava operator $T_{\sigma }^{n}$ if ς=0 ^15, 16^
c) Salagean ^17^ examined the case if μ=1,ς=−n,n=1,σ=1 .
d) It was examined by Salagean ^17^ and Flett ^18^ if μ=1,ς=n,n=1,σ=1 .

Definition 2.4. If σ∈ℕ,0≤π<1,μ>0;n,ς≥0, then let $R_{\sigma ,\mu }^{n,\varsigma }\left(\pi \right)$ denote the class of a function ϒ∈Λ that satisfies the given conditions.

$$R{\left(M_{\sigma ,\mu }^{n,\varsigma }ϒ\left(s\right)\right)}^{\prime }>\pi ,\left(s\;\in \;\Delta^{*}\right).$$ (8)

Lemma 2.1. ^19^ Assume that ϒ is in Λ, if $R\left(1+\frac{s{ϒ}^{\prime \prime }\left(s\right)}{{ϒ}^{\prime }\left(s\right)}\right)>-\frac{1}{2},$ then

$$\frac{2}{s}\int_{0}^{s}ϒ\left(\tau \right)\mathrm{d\tau},\left(s\;\in \;\Delta^{*}\;\text{and}\;s\neq 0\right),$$

is a convex functions.

Lemma 2.2. ^20^ Let ϕ be a convex function in $\;\Delta^{*}$ such that

$\psi \left(s\right)=\phi \left(s\right)+\mathrm{\eta \beta s}\phi^{\prime }\left(s\right),\left(s\;\in \;\Delta^{*}\right),$ in which β>0 and η∈ℕ. If $\varpi \left(s\right)=\phi \left(0\right)+\varpi_{\eta }s^{\eta }+\varpi_{\eta +1}s^{\eta +1}+\ldots ,$ is analytic in $\Delta^{*}\;$ and $\varpi \left(s\right)+\mathrm{\beta s}\varpi^{\prime }\left(s\right)\;≺\;\psi \left(s\right)$ , then

ϖ(s)≺ϕ(s).

Lemma 2.3. ^19^ Consider the following ψ(0)=e , ξ is analytic, univalent, and convex in Δ, γ∈ℂ∖{0} is a complex number that produces R{γ}≥0. Given ϖ∈Η[e,η] as well as $\varpi \left(s\right)+\frac{s\varpi^{\prime }\left(s\right)}{\gamma }\;≺\;\psi \left(s\right),$ then

$$\varpi \left(s\right)\;≺\;\xi \left(s\right)\;≺\;\psi \left(s\right),\;\left(s\;\in \;\Delta^{*}\right),\text{where}\;\;\xi \left(s\right)=\frac{\gamma }{\eta s^{\frac{\gamma }{\eta }}}\int_{0}^{s}\;\psi \left(\tau \right)\tau^{\frac{\gamma }{\eta }-1}\mathrm{d\tau},\;\left(s\;\in \;\Delta^{*}\right).$$

The optimal prevailing of the subordination is the convex function ξ .

## 3. Results and Discussion

In this study, we will derive multiple characteristics derived from the new linear operator $\;M_{\sigma ,\mu }^{n,\varsigma }ϒ\left(s\right)$ using differential subordination technique.

Theorem 3.1. Let ψ(0)=1,0≤π<1 and $\;\psi \left(s\right)=\frac{1+\left(2\pi -1\right)s}{1+s}$ be convex in $\Delta^{*}$ . Suits the differential subordination ${\left(M_{\sigma ,\mu }^{n+1,\varsigma }ϒ\left(s\right)\right)}^{\prime }\;≺\;\psi \left(s\right)$ for σ∈ℕ,μ>0;n,ς≥0 as well as ϒ∈A , then

$${\left(M_{\sigma ,\mu }^{n,\varsigma }ϒ\left(s\right)\right)}^{\prime }\;≺\;\xi \left(s\right)=\left(\frac{3}{2\pi }-1\right)+3/2\frac{\left(\beta_{1}\mu +1\right)\left(1-\pi \right)}{\eta s^{\left(\beta_{1}\mu +1\right)/\eta }}\;\zeta \left(\left(\beta_{1}\mu +1\right)/\eta \right),$$ (9)

where

$$\;\zeta \left(x\right)=\int_{0}^{s}\frac{\tau^{x-1}}{1+\tau }\mathrm{d\tau},\left(s\;\in \;\Delta^{*}\right).$$

Proof. The result of differentiation Eq. (7) is

$$\begin{array}{c} \left(\beta_{1}\mu +1\right){\left(M_{\sigma ,\mu }^{n+1,\varsigma }ϒ\left(s\right)\right)}^{\prime }=\left(\beta_{1}\mu +1\right){\left(M_{\sigma ,\mu }^{n,\varsigma }ϒ\left(s\right)\right)}^{\prime }+s{\left(M_{\sigma ,\mu }^{n,\varsigma }ϒ\left(s\right)\right)}^{\prime \prime }\\ {\left(M_{\sigma ,\mu }^{n+1,\varsigma }ϒ\left(s\right)\right)}^{\prime }=\frac{\left(\beta_{1}\mu +1\right){\left(M_{\sigma ,\mu }^{n,\varsigma }ϒ\left(s\right)\right)}^{\prime }+s{\left(M_{\sigma ,\mu }^{n,\varsigma }ϒ\left(s\right)\right)}^{\prime \prime }}{\left(\beta_{1}\mu +1\right)}\;\\ {\left(M_{\sigma ,\mu }^{n+1,\varsigma }ϒ\left(s\right)\right)}^{\prime }={\left(M_{\sigma ,\mu }^{n,\varsigma }ϒ\left(s\right)\right)}^{\prime }+\frac{s{\left(M_{\sigma ,\mu }^{n,\varsigma }ϒ\left(s\right)\right)}^{\prime \prime }}{\left(\beta_{1}\mu +1\right)}. \end{array}$$ (10)

When Eq. (10) is used in Eq. (9), the subordination Eq. (9) is transformed into

$${\left(M_{\sigma ,\mu }^{n,\varsigma }ϒ\left(s\right)\right)}^{\prime }+\frac{s{\left(M_{\sigma ,\mu }^{n,\varsigma }ϒ\left(s\right)\right)}^{\prime \prime }}{\left(\beta_{1}\mu +1\right)}\;≺\;\psi \left(s\right)=\frac{1+\left(\frac{3}{2\pi }-1\right)}{1+s}.$$ (11)

Let

$$\begin{array}{c} \varpi \left(s\right)={\left(M_{\sigma ,\mu }^{n,\varsigma }ϒ\left(s\right)\right)}^{\prime }={\left(s+\sum_{\eta =2}^{\infty }\frac{{\left(\beta_{1}\mu^{\varsigma }\right)}_{\eta -1}{\left(\beta_{2}\right)}_{\eta -1}\ldots {\left(\beta_{n}\right)}_{\eta -1}}{{\left(\pi_{1}\right)}_{\eta -1}{\left(\pi_{2}\right)}_{\eta -1}\ldots {\left(\pi_{\sigma }\right)}_{\eta -1}}{\left(\frac{1}{\mu +\eta -1}\right)}^{-\varsigma +1}e_{\eta }s^{\eta }\;\right)}^{\prime },\\ \varpi \left(s\right)=1+\varpi_{1}s+\varpi_{2}s^{2}+\ldots ,\;\varpi \;\in \;Η\left[1,1\right],s\;\in \;\Delta^{*}. \end{array}$$ (12)

Subordination is made possible in Eq. (11), through the use of Eq. (12).

$$\varpi \left(s\right)+\frac{s\varpi^{\prime }\left(s\right)}{\left(\beta_{1}\mu +1\right)}\;≺\;\psi \left(s\right)=\frac{1+\left(3/2\pi -1\right)}{1+s}.$$

Employing Lemma 2.3, has been

$$\begin{array}{c} \varpi \left(s\right)\;≺\;\xi \left(s\right)=\frac{\left(\beta_{1}\mu +1\right)}{\eta s^{\left(\beta_{1}\mu +1\right)∕\eta }}\int_{0}^{s}\;\psi \left(\tau \right)\tau^{\left(\left(\beta_{1}\mu +1\right)/\eta \right)-1}\mathrm{d\tau}\\ =\frac{\left(\beta_{1}\mu +1\right)}{\eta s^{\left(\beta_{1}\mu +1\right)∕\eta }}\int_{0}^{s}\left[\left(3/2\pi -1\right)+3/2\left(1-\pi \right)\frac{1}{1+\tau }\right]\frac{\tau^{\left(\left(\beta_{1}\mu +1\right)/\eta \right)-1}}{1+\tau }\mathrm{d\tau}\\ \begin{array}{c} =\frac{\left(\beta_{1}\mu +1\right)}{\eta s^{\left(\beta_{1}\mu +1\right)/\eta }}\int_{0}^{s}\left(3/2\pi -1\right)\tau^{\left(\left(\beta_{1}\mu +1\right)/\eta \right)-1}\mathrm{d\tau}+\frac{3/2\left(1-\pi \right)\left(\beta_{1}\mu +1\right)}{\eta s^{\left(\beta_{1}\mu +1\right)∕\eta }}\int_{0}^{s}\frac{\tau^{\left(\left(\beta_{1}\mu +1\right)/\eta \right)-1}}{1+\tau }\mathrm{d\tau}\\ =\left(3/2\pi -1\right)+3/2\frac{\left(\beta_{1}\mu +1\right)\left(1-\pi \right)}{\eta s^{\left(\beta_{1}\mu +1\right)∕\eta }}\;\zeta \left(\left(\beta_{1}\mu +1\right)∕\eta \right), \end{array} \end{array}$$

then

$${\left(M_{\sigma ,\mu }^{n,\varsigma }ϒ\left(s\right)\right)}^{\prime }\;≺\;\xi \left(s\right)=\left(3/2\pi -1\right)+3/2\frac{\left(\beta_{1}\mu +1\right)\left(1-\pi \right)}{\eta s^{\left(\beta_{1}\mu +1\right)/\eta }}\;\zeta \left(\left(\beta_{1}\mu +1\right)/\eta \right).$$

Theorem 3.2. Given that ξ is a convex function in $\Delta^{*}$ with ξ(0)=1 and $\;\psi \left(s\right)=\xi \left(s\right)+s\;\xi^{\prime }\left(s\right).$ If ϒ∈Λ , σ∈N,μ>0;n,ς≥0,0≤π<1 satisfies the subordination $\;{\left(M_{\sigma ,\mu }^{n,\varsigma }ϒ\left(s\right)\right)}^{\prime }\;≺\;\psi \left(s\right),$ then

$$\frac{M_{\sigma ,\mu }^{n,\varsigma }ϒ\left(s\right)}{s}\;≺\;\xi \left(s\right),\;\left(s\;\in \;\Delta^{*}\right).$$ (13)

Proof. Let $\varpi \left(s\right)=\frac{M_{\sigma ,\mu }^{n,\varsigma }ϒ\left(s\right)}{s}$

$$\;\begin{array}{c} \varpi \left(s\right)=\frac{s+\sum_{\eta =2}^{\infty }\frac{{\left(\beta_{1}\mu^{\varsigma }\right)}_{\eta -1}{\left(\beta_{2}\right)}_{\eta -1}\ldots {\left(\beta_{n}\right)}_{\eta -1}}{{\left(\pi_{1}\right)}_{\eta -1}{\left(\pi_{2}\right)}_{\eta -1}\ldots {\left(\pi_{\sigma }\right)}_{\eta -1}}{\left(\frac{1}{\mu +\eta -1}\right)}^{-\varsigma +1}e_{\eta }s^{\eta }\;}{s},\\ \varpi \left(s\right)=1+\sum_{\eta =2}^{\infty }\frac{{\left(\beta_{1}\mu^{\varsigma }\right)}_{\eta -1}{\left(\beta_{2}\right)}_{\eta -1}\ldots {\left(\beta_{n}\right)}_{\eta -1}}{{\left(\pi_{1}\right)}_{\eta -1}{\left(\pi_{2}\right)}_{\eta -1}\ldots {\left(\pi_{\sigma }\right)}_{\eta -1}}{\left(\frac{1}{\mu +\eta -1}\right)}^{-\varsigma +1}e_{\eta }s^{\eta -1}\;\\ \begin{array}{c} \varpi \left(s\right)=1+\varpi_{\eta }s^{\eta }+\varpi_{\eta +1}s^{\eta +1}+\ldots \\ {\left(M_{\sigma ,\mu }^{n,\varsigma }ϒ\left(s\right)\right)}^{\prime }=\varpi \left(s\right)+s\varpi^{\prime }\left(s\right). \end{array} \end{array}$$

Consequently, with the help of the connection Eq. (13) grows

$$\varpi \left(s\right)+s\varpi^{\prime }\left(s\right)\;≺\;\psi \left(s\right)=\;\xi \left(s\right)+s\;\xi^{\prime }\left(s\right).$$

Utilizing Lemma 2.2, previously

ϖ(s)≺ξ(s).

Through the use of $\varpi \left(s\right)=\frac{M_{\sigma ,\mu }^{n,\varsigma }ϒ\left(s\right)}{s}$ , we obtain

$$\frac{M_{\sigma ,\mu }^{n,\varsigma }ϒ\left(s\right)}{s}\;≺\;\xi \left(s\right).$$

Theorem 3.3. If σ∈N,μ>0;n,ς≥0 as well as 0≤π<1, then $R_{\sigma ,\mu }^{n+1,\varsigma }\left(\pi \right)\subset R_{\sigma ,\mu }^{n,\varsigma }\left(\beta \right)$ such that

$$\varepsilon =\left(3/2\pi -1\right)+3/2\left(1-\pi \right)\frac{\left(\beta_{1}\mu +1\right)}{\eta }\zeta \left(\left(\beta_{1}\mu +1\right)/\eta \right),\;\zeta =\int_{0}^{s}\frac{\tau^{x-1}}{1+\tau }\mathrm{d\tau}.$$ (14)

Proof. Assume ϒ is in $ϒ\;\in \;R_{\sigma ,\mu }^{n+1,\varsigma }\left(\pi \right).$ Next, based on Eq. (8), there is $R{\left(M_{\sigma ,\mu }^{n,\varsigma }ϒ\left(s\right)\right)}^{\prime }>\pi$ this is equivalent to

$${\left(M_{\sigma ,\mu }^{n+1,\varsigma }ϒ\left(s\right)\right)}^{\prime }\;≺\;\psi \left(s\right)=\frac{1+\left(3/2\pi -1\right)s}{1+s}.\;$$

Applying Theorem 3.1, we arrive at

$${\left(M_{\sigma ,\mu }^{n,\varsigma }ϒ\left(s\right)\right)}^{\prime }\;≺\;\xi \left(s\right)=\left(3/2\pi -1\right)+3/2\frac{\left(\beta_{1}\mu +1\right)\left(1-\pi \right)}{\eta s^{\left(\beta_{1}\mu +1\right)/\eta }}\;\zeta \left(\left(\beta_{1}\mu +1\right)/\eta \right).$$

Since $\;\xi \left(\Delta^{*}\right)$ is symmetric when compared to the real direction and ξ is convex, we are able to deduce that

Re(Mσ,μn,ςϒ(s))′>Reξ(1)=ε=ε(π,β1,μ,η)=(3/2π−1)+3/2(β1μ+1)(1−π)ηζ((β1μ+1)/η),

where

$$\begin{array}{c} \;\xi \left(1\right)=\frac{\left(\beta_{1}\mu +1\right)}{\eta }\int_{0}^{1}\left[\frac{1+\left(3/2\pi -1\right)\tau }{1+\tau }\right]\tau^{\left(\left(\beta_{1}\mu +1\right)/\eta \right)-1}\mathrm{d\tau}\\ =\frac{\left(\beta_{1}\mu +1\right)}{\eta }\int_{0}^{1}\left[\left(3/2\pi -1\right)+3/2\left(1-\pi \right)\frac{1}{1+\tau }\right]\frac{\tau^{\left(\left(\beta_{1}\mu +1\right)/\eta \right)-1}}{1+\tau }\mathrm{d\tau}\\ \begin{array}{c} =\frac{\left(\beta_{1}\mu +1\right)}{\eta }\int_{0}^{1}\left(3/2\pi -1\right)\tau^{\left(\left(\beta_{1}\mu +1\right)/\eta \right)-1}\mathrm{d\tau}+\frac{3/2\left(1-\pi \right)\left(\beta_{1}\mu +1\right)}{\eta }\int_{0}^{1}\frac{\tau^{\left(\left(\beta_{1}\mu +1\right)/\eta \right)-1}}{1+\tau }\mathrm{d\tau}\\ =\left(3/2\pi -1\right)+3/2\frac{\left(\beta_{1}\mu +1\right)\left(1-\pi \right)}{\eta }\;\zeta \left(\left(\beta_{1}\mu +1\right)/\eta \right) \end{array} \end{array}$$

It leads us to the conclusion that $R_{\sigma ,\mu }^{n+1,\varsigma }\left(\pi \right)\subset R_{\sigma ,\mu }^{n,\varsigma }\left(\beta \right).$ The evidence is finished.

Theorem 3.4. Let ψ be a convex function in $\Delta^{*}$ , ψ(0)=1,0≤π<1, and $\;\psi \left(s\right)=\frac{1+\left(3/2\pi -1\right)s}{1+s}.$ If σ∈ℕ,μ>0;n,ς≥0,ϒ∈Λ satisfies the subordination ${\left(M_{\sigma ,\mu }^{n,\varsigma }ϒ\left(s\right)\right)}^{\prime }\;≺\;\psi \left(s\right),$ then

$$\frac{M_{\sigma ,\mu }^{n,\varsigma }ϒ\left(s\right)}{s}\;≺\;\xi \left(s\right)=\left(3/2\pi -1\right)+\frac{3/2\left(1-\pi \right)\ln\left(1+s\right)}{s},\;\left(s\;\in \;\Delta^{*}\right).$$

The function ξ is convex as well as the most prevailing.

Proof. Suppose

$$\begin{array}{c} \varpi \left(s\right)=\frac{M_{\sigma ,\mu }^{n,\varsigma }ϒ\left(s\right)}{s}=\frac{\;s+\sum_{\eta =2}^{\infty }\frac{{\left(\beta_{1}\mu^{\varsigma }\right)}_{\eta -1}{\left(\beta_{2}\right)}_{\eta -1}\ldots {\left(\beta_{n}\right)}_{\eta -1}}{{\left(\pi_{1}\right)}_{\eta -1}{\left(\pi_{2}\right)}_{\eta -1}\ldots {\left(\pi_{\sigma }\right)}_{\eta -1}}{\left(\frac{1}{\mu +\eta -1}\right)}^{-\varsigma +1}e_{\eta }s^{\eta }\;}{s},\\ =\varpi \left(s\right)=1+\varpi_{\eta }s^{\eta }+\varpi_{\eta +1}s^{\eta +1}+\ldots . \end{array}$$ (15)

By Eq. (15) in relation to ϖ , we get

$${\left(M_{\sigma ,\mu }^{n,\varsigma }ϒ\left(s\right)\right)}^{\prime }=\varpi \left(s\right)+s\varpi^{\prime }\left(s\right),\;\left(s\;\in \;\Delta^{*}\right).$$ (16)

By applying Eq. (16), differential subordination ${\left(M_{\sigma ,\mu }^{n,\varsigma }ϒ\left(s\right)\right)}^{\prime }\;≺\;\psi \left(s\right),$ is obtained

$$\varpi \left(s\right)+s\varpi^{\prime }\left(s\right)\;≺\;\psi \left(s\right)=\frac{1+\left(3/2\pi -1\right)s}{1+s},\;\left(s\;\in \;\Delta^{*}\right).\;$$

Utilizing Lemma 2.3, has been

$$\begin{array}{c} \varpi \left(s\right)\;≺\;\xi \left(s\right)=\frac{1}{s}\int_{0}^{s}\;\psi \left(\tau \right)\mathrm{d\tau}=\frac{1}{s}\int_{0}^{s}\left(\frac{1+\left(3/2\pi -1\right)\tau }{1+\tau }\right)\mathrm{d\tau}\\ =\frac{1}{s}\int_{0}^{s}\left[\left(3/2\pi -1\right)+3/2\left(1-\pi \right)\frac{1}{1+\tau }\right]\mathrm{d\tau}\;\\ \begin{array}{c} =\frac{1}{s}\int_{0}^{s}\left(3/2\pi -1\right)\mathrm{d\tau}+\frac{3/2\left(1-\pi \right)}{s}\int_{0}^{s}\frac{1}{1+\tau }\mathrm{d\tau}\\ =\left(3/2\pi -1\right)+\frac{3/2\left(1-\pi \right)\ln\left(1+s\right)}{s}. \end{array} \end{array}$$

With the help of Eq. (15), we had

$$\frac{M_{\sigma ,\mu }^{n,\varsigma }ϒ\left(s\right)}{s}\;≺\;\xi \left(s\right)=\left(3/2\pi -1\right)+\frac{3/2\left(1-\pi \right)\ln\left(1+s\right)}{s}.$$

Theorem 3.4, has been fully proved.

Theorem 3.5. If ψ is convex function in $\Delta^{*}$ and $\;\psi \left(0\right)=1,\;0\;\leq \;\pi <1,\;\psi \left(s\right)=\frac{1+\left(3/2\pi -1\right)s}{1+s},$ ϒ∈Λ,σ∈ℕ,κ>0;n,ς≥0 satisfies the subordination

$$1-\frac{M_{\sigma ,\mu }^{n,\varsigma }ϒ\left(s\right){\left(M_{\sigma ,\mu }^{n,\varsigma }ϒ\left(s\right)\right)}^{\prime \prime }}{{\left[{\left(M_{\sigma ,\mu }^{n,\varsigma }ϒ\left(s\right)\right)}^{\prime }\right]}^{2}}\;≺\;\psi \left(s\right),$$ (17)

then

$$\frac{M_{\sigma ,\mu }^{n,\varsigma }ϒ\left(s\right)}{s{\left(M_{\sigma ,\mu }^{n,\varsigma }ϒ\left(s\right)\right)}^{\prime }}\;≺\;\xi \left(s\right)=\left(3/2\pi -1\right)+\frac{3/2\left(1-\pi \right)\ln\left(1+s\right)}{s}.$$

Proof. Suppose

$$\begin{array}{c} \varpi \left(s\right)=\frac{M_{\sigma ,\mu }^{n,\varsigma }ϒ\left(s\right)}{s{\left(M_{\sigma ,\mu }^{n,\varsigma }ϒ\left(s\right)\right)}^{\prime }},\\ \varpi \left(s\right)=\frac{s+\sum_{\eta =2}^{\infty }\frac{{\left(\beta_{1}\mu^{\varsigma }\right)}_{\eta -1}{\left(\beta_{2}\right)}_{\eta -1}\ldots {\left(\beta_{n}\right)}_{\eta -1}}{{\left(\pi_{1}\right)}_{\eta -1}{\left(\pi_{2}\right)}_{\eta -1}\ldots {\left(\pi_{\sigma }\right)}_{\eta -1}}{\left(\frac{1}{\mu +\eta -1}\right)}^{-\varsigma +1}e_{\eta }s^{\eta }\;}{s\left(1+\sum_{\eta =2}^{\infty }\frac{{\left(\beta_{1}\mu^{\varsigma }\right)}_{\eta -1}{\left(\beta_{2}\right)}_{\eta -1}\ldots {\left(\beta_{n}\right)}_{\eta -1}}{{\left(\pi_{1}\right)}_{\eta -1}{\left(\pi_{2}\right)}_{\eta -1}\ldots {\left(\pi_{\sigma }\right)}_{\eta -1}}{\left(\frac{1}{\mu +\eta -1}\right)}^{-\varsigma +1}e_{\eta }s^{\eta -1}\;\right)}, \end{array}$$

so that with the help of Eq. (17) becomes

$$\varpi \left(s\right)+s\varpi^{\prime }\left(s\right)\;≺\;\psi \left(s\right)=\frac{1+\left(3/2\pi -1\right)s}{1+s},\;\left(s\;\in \;\Delta^{*}\right).\;$$

By Lemma 2.3 allows us to have

$$\begin{array}{c} \varpi \left(s\right)\;≺\;\xi \left(s\right)=\frac{1}{s}\int_{0}^{s}\;\psi \left(\tau \right)\mathrm{d\tau}=\frac{1}{s}\int_{0}^{s}\left(\frac{1+\left(3/2\pi -1\right)\tau }{1+\tau }\right)\mathrm{d\tau}\\ =\frac{1}{s}\int_{0}^{s}\left[\left(3/2\pi -1\right)+3/2\left(1-\pi \right)\frac{1}{1+\tau }\right]\mathrm{d\tau}=\left(3/2\pi -1\right)+\frac{3/2\left(1-\pi \right)\ln\left(1+s\right)}{s}, \end{array}$$

then

$$\frac{M_{\sigma ,\mu }^{n,\varsigma }ϒ\left(s\right)}{s{\left(M_{\sigma ,\mu }^{n,\varsigma }ϒ\left(s\right)\right)}^{\prime }}\;≺\;\xi \left(s\right)=\left(3/2\pi -1\right)+\frac{3/2\left(1-\pi \right)\ln\left(1+s\right)}{s}.$$

Theorem 3.6. If ϒ∈Λ and suppose that ξ is an analytic function that fulfills the next inequality $R\left\{1+\frac{s\;\psi^{\prime \prime }\left(s\right)}{\;\psi^{\prime }\left(s\right)}\right\}>-\frac{1}{2},$ and $\;\psi \left(0\right)=1,\psi^{\prime }\left(0\right)\neq 0,\sigma \;\in \;N,\mu >0;n,\varsigma \;\geq \;0\;$ satisfies the subordination

$${\left(M_{\sigma ,\mu }^{n,\varsigma }ϒ\left(s\right)\right)}^{\prime }\;≺\;\psi \left(s\right),\;\left(s\;\in \;\Delta^{*}\right),$$ (18)

then

$$\frac{M_{\sigma ,\mu }^{n,\varsigma }ϒ\left(s\right)}{s}\;≺\;\xi \left(s\right)=\frac{1}{s}\int_{0}^{s}\;\psi \left(\tau \right)\mathrm{d\tau}.$$

Proof. Suppose

$$\begin{array}{c} \varpi \left(s\right)=\frac{M_{\sigma ,\mu }^{n,\varsigma }ϒ\left(s\right)}{s}=\frac{s+\sum_{\eta =2}^{\infty }\frac{{\left(\beta_{1}\mu^{\varsigma }\right)}_{\eta -1}{\left(\beta_{2}\right)}_{\eta -1}\ldots {\left(\beta_{n}\right)}_{\eta -1}}{{\left(\pi_{1}\right)}_{\eta -1}{\left(\pi_{2}\right)}_{\eta -1}\ldots {\left(\pi_{\sigma }\right)}_{\eta -1}}{\left(\frac{1}{\mu +\eta -1}\right)}^{-\varsigma +1}e_{\eta }s^{\eta }\;}{s},\\ \varpi \left(s\right)=1+\varpi_{\eta }s^{\eta }+\varpi_{\eta +1}s^{\eta +1}+\ldots . \end{array}$$ (19)

By Eq. (19) in relation to w, we get

$${\left(M_{\sigma ,\mu }^{n,\varsigma }ϒ\left(s\right)\right)}^{\prime }=\varpi \left(s\right)+s\varpi^{\prime }\left(s\right),\;\left(s\;\in \;\Delta^{*}\right).$$ (20)

Subordination Eq. (18), when applied to Eq. (20), grows

$$\varpi \left(s\right)+s\varpi^{\prime }\left(s\right)\;≺\;\psi \left(s\right).$$

By Lemma 2.3 allows us to have

ϖ(s)≺ξ(s),

in other words,

$$\frac{M_{\sigma ,\mu }^{n,\delta }ϒ\left(s\right)}{s}\;≺\;\xi \left(s\right)=\frac{1}{s}\int_{0}^{s}\;\psi \left(\tau \right)\mathrm{d\tau}.$$

Theorem 3.7. If ϒ∈Λ and suppose ξ be convex function in $\Delta^{*}$ *,* ξ(0)=1 and $\;\psi \left(s\right)=\xi \left(s\right)+s\;\xi^{\prime }\left(s\right)$ , σ∈N,μ>0;n,ς≥0 , 0≤π<1 satisfies subordination

$$1-\frac{M_{\sigma ,\mu }^{n,\varsigma }ϒ\left(s\right){\left(M_{\sigma ,\mu }^{n,\varsigma }ϒ\left(s\right)\right)}^{\prime \prime }}{{\left[{\left(M_{\sigma ,\mu }^{n,\varsigma }ϒ\left(s\right)\right)}^{\prime }\right]}^{2}}\;≺\;\psi \left(s\right),\;\left(s\;\in \;\Delta^{*}\right),$$ (21)

then

$$\frac{M_{\sigma ,\mu }^{n,\varsigma }ϒ\left(s\right)}{s{\left(M_{\sigma ,\mu }^{n,\varsigma }ϒ\left(s\right)\right)}^{\prime }}\;≺\;\xi \left(s\right).$$ (22)

Proof. Suppose

$$\varpi \left(s\right)=\frac{M_{\sigma ,\mu }^{n,\varsigma }ϒ\left(s\right)}{s{\left(M_{\sigma ,\mu }^{n,\varsigma }ϒ\left(s\right)\right)}^{\prime }},\;p\;\in \;H\left[1,1\right].$$

Taking the product of both perspectives gives us

$$\varpi \left(s\right)+s\varpi^{\prime }\left(s\right)=1-\frac{M_{\sigma ,\mu }^{n,\varsigma }ϒ\left(s\right){\left(M_{\sigma ,\mu }^{n,\varsigma }ϒ\left(s\right)\right)}^{\prime \prime }}{{\left[{\left(M_{\sigma ,\mu }^{n,\varsigma }ϒ\left(s\right)\right)}^{\prime }\right]}^{2}},$$

so that with the help of Eq. (21) becomes

$$\varpi \left(s\right)+s\varpi^{\prime }\left(s\right)\;≺\;\psi \left(s\right)=\xi \left(s\right)+s\;\xi^{\prime }\left(s\right),\;\left(s\;\in \;\Delta^{*}\right).\;$$

By Lemma 2.2, we obtain

ϖ(s)≺ξ(s),

then

$$\frac{M_{\sigma ,\mu }^{n,\varsigma }ϒ\left(s\right)}{s{\left(M_{\sigma ,\mu }^{n,\varsigma }ϒ\left(s\right)\right)}^{\prime }}\;≺\;\xi \left(s\right).$$

## 4. Conclusions

There are many fascinating discoveries regarding harmonic multivalent functions derived from differential operators. The study concentrated on a subclass of analytical univalent functions related to the concept of differential subordination. Everyone looked at a few differential subordination and superordination outcomes, such as a class determined by a dimension for univalent meromorphic functions inside the open unit disc. Learn about geometrical characteristics such as coefficient border, coefficient disparities, distortion theorem, closing theorem, severe points, starlikeness radii, convexity, near-perfect convexity, and combining principles. An analysis is conducted on the concept of the differential subordination subclass of analytical univalent functions. Utilizing a particular class of univalent functions stated on a particular space of univalent functions stated on the open unit disc, we examined certain findings on differential subordination as well as superordination. We found several properties of subordinations as well as superordinations connected to the notion of Hadamard product by using characteristics of the operator. We examined various facets of subordinations as well as superordinations through a novel operator, $M_{\sigma ,\mu }^{n,\varsigma }ϒ\left(s\right)$ .

## Data Availability

Data sharing is not applicable to this article, as no data were created or analyzed in this study.

## References

[1] Littlewood JE : *Lectures on the Theory of Functions.* UK: Oxford University Press;1944;244.

[2] Rogosinski W : On subordination functions. *Proc. Camb. Philos. Soc.* 1939;35:1–26. 10.1017/S0305004100020703

[3] Srivastava HM Owa S : Some Applications of the Generalized Hypergeometric Function Involving Certain Subclasses of Analytic Functions. *Publ. Math. Debr.* 1987;34(3-4):299–306. 10.5486/PMD.1987.34.3-4.13

[4] Miller SS Mocanu PT : Second-order Differential Inequalities in the Complex Plane. *J. Math. Anal. Appl.* 1978;65(2):289–305. 10.1016/0022-247X(78)90181-6

[5] Cotîrla L Catas A : Differential sandwich theorem for certain class of analytic functions associated with an integral operator. *Stud. Univ. Babes-Bolyai Math.* 2020;65:487–494. 10.24193/subbmath.2020.4.01

[6] Hameed MI Ali MH Shihab BN : A Certain Subclass of Meromorphically Multivalent Q-Starlike Functions Involving Higher-Order Q-Derivatives. *Iraqi Journal of Science.* 2022;63(1):251–258. 10.24996/ijs.2022.63.1.26

[7] Mazı E Altınkaya S : On a new subclass of bi-univalent functions satisfying subordinate conditions. *Communications Faculty of Sciences University of Ankara Series A1 Mathematics and Statistics.* 2019;68(1):724–733. 10.31801/cfsuasmas.464191

[8] Badriev IB Bujanov VY Makarov MV : Differential properties of the operator of the geometrically nonlinear problem of a sandwich plate bending. *Lobachevskii Journal of Mathematics.* 2019;40:263–273.

[9] Hameed MI Shihab BN : Several subclasses of r-fold symmetric bi-univalent functions possess coefficient bounds. *Iraqi Journal of Science.* 2022;63(12):5438–5446. 10.24996/ijs.2022.63.12.29

[10] Al-Dulaimi SJ Hameed MI Hameed II : Results of third order differential subordination for holomorphic functions. *Journal of Interdisciplinary Mathematics.* 2024;27(4):881–886. 10.47974/JIM-1907

[11] Reddy DM : Amalgamative Application of Derivative Operator and Differential Sub-Ordination in Developing Theorems. *Turkish Journal of Computer and Mathematics Education(TURCOMAT).* 2021;12(6):166–171. 10.17762/turcomat.v12i6.1282

[12] Shehab NH Juma ARS : Third Order Differential Subordination for Analytic Functions Involving Convolution Operator. *Baghdad Sci. J.* 2022;19(3):0581–0581. 10.21123/bsj.2022.19.3.0581

[13] Frasin BA Yousef F Al-Hawary T : Application of generalized Bessel functions to classes of analytic functions. *Afr. Mat.* 2021;32:431–439. 10.1007/s13370-020-00835-9

[14] Komatu Y : On distortion properties of analytic operators. *Kodai mathematical journal.* 1992;15(1):1–10. 10.2996/kmj/1138039523

[15] Alb LA : New Results on a Fractional Integral of Extended Dziok–Srivastava Operator Regarding Strong Subordinations and Superordinations. *Symmetry.* 2023;15(8):1544. 10.3390/sym15081544

[16] Salagean G : Subclasses of univalent functions. *Complex analysis Fifth Romanian Finnish Seminar, Part 1 (Bucharest, 1981) of Lecture Notes in Mathematics.* Berlin. Germany: Springer;1983;1013: p.362–372.

[17] Paulino D : Critical Hardy–Littlewood inequality for multilinear forms. *Rendiconti del Circolo Matematico di Palermo Series.* 2020;69(2):369–380. 10.1007/s12215-019-00409-0

[18] Domelevo K Petermichl S : Differential subordination under change of law. *Ann. Probab.* 2019;47(2):896–925. 10.1214/18-AOP1274

[19] Miller SS Mocanu PT : *Differential Subordinations. Theory and Applications of Monographs and Textbooks in Pure and Applied Mathematics.* New York, NY, USA: Marcel Dekker;2000.

[20] Kumar SS Banga S : On Convex Dominants of Exact Differential Subordination. *arXiv preprint arXiv.* 2020;11404. 2011.

