# Supplementary material for: Study analytical function subordination properties by applying a novel linear operator
Source: F1000Res. 2025 Dec 31;14:1479. [Version 1] doi: 10.12688/f1000research.174492.1 (PMC12829319; doi:10.12688/f1000research.174492.1)
Supplement: Peer review history

# Reviewer report: Reviewer response for version 1

**DOI:** 10.5256/f1000research.192398.r447074

**Published:** 2026-01-16

**Authors:** Kassim A. Jassim

**Affiliations:** 1 University of Baghdad, Baghdad, Baghdad Governorate, Iraq

The paper is studied some results on differential subordination and superordination using a class of univalent functions defined on the open unit disc. Many properties of the of subordinations and superordinations are investigated , using new operator and it's identity .Also , cited references is really used in paper and many of these are modern which make paper more specified and important.

Is the work clearly and accurately presented and does it cite the current literature?

Yes

If applicable, is the statistical analysis and its interpretation appropriate?

Yes

Are all the source data underlying the results available to ensure full reproducibility?

Yes

Is the study design appropriate and is the work technically sound?

Yes

Are the conclusions drawn adequately supported by the results?

Yes

Are sufficient details of methods and analysis provided to allow replication by others?

Yes

Reviewer Expertise:

Complex analysis

I confirm that I have read this submission and believe that I have an appropriate level of expertise to confirm that it is of an acceptable scientific standard.

# Reviewer report: Reviewer response for version 1

**DOI:** 10.5256/f1000research.192398.r447076

**Published:** 2026-01-07

**Authors:** Alina Alb Lupas

**Affiliations:** 1 University of Oradea, Oradea, Romania

**REVIEWER’s REPORT**

*On the paper*

 Study analytical function subordination properties by applying a novel linear operator

 by Maryam S. Majel and Mustafa I. Hameed.

 In this paper the authors define and study a new operator by using the Dziok-Srivastava operator and the Hadamard product with the Komatu integral operator. They establish several properties of subordinations and superordinations regarding the new defined operator.

The results are new, correct and detailed. The paper is original and doesn’t contradict to ethical or policy issues, the question posed by authors is new and well defined, the methods used by authors are appropriate and well described, the data are sound and well controlled, the discussion and conclusions are well balanced, the title and abstract convey the obtained results, the writing is acceptable, the paper contains good scientific results.

The paper doesn’t require a revision.

Taking the above into consideration, I recommend the paper for indexing.

"No competing interests were disclosed"

Is the work clearly and accurately presented and does it cite the current literature?

Yes

If applicable, is the statistical analysis and its interpretation appropriate?

Not applicable

Are all the source data underlying the results available to ensure full reproducibility?

Yes

Is the study design appropriate and is the work technically sound?

Yes

Are the conclusions drawn adequately supported by the results?

Yes

Are sufficient details of methods and analysis provided to allow replication by others?

Yes

Reviewer Expertise:

Complex analysis

I confirm that I have read this submission and believe that I have an appropriate level of expertise to confirm that it is of an acceptable scientific standard.
